# Students’ attitudes and perceptions of teaching and assessment of evidence-based practice in an occupational therapy professional Master’s curriculum: a mixed methods study

**DOI:** 10.1186/s12909-017-0895-2

**Published:** 2017-03-27

**Authors:** Aliki Thomas, Lu Han, Brittony P. Osler, Emily A. Turnbull, Erin Douglas

**Affiliations:** 1 School of Physical and Occupational Therapy, 3654 Sir William Osler, Montréal, Québec H3G 1Y5 Canada; 2 Research Scientist, Center for Medical Education, Faculty of Medicine, McGill University, 1110 Pine Avenue West, Montréal, Québec H3G 1A3 Canada; 3 Centre for Interdisciplinary Research in Rehabilitation, Montréal, Québec Canada

**Keywords:** Evidence-based practice, Rehabilitation, Curriculum, Professional education, Attitudes, Self-efficacy

## Abstract

**Background:**

Most health professions, including occupational therapy, have made the application of evidence-based practice a desired competency and professional responsibility. Despite the increasing emphasis on evidence-based practice for improving patient outcomes, there are numerous research-practice gaps in the health professions. In addition to efforts aimed at promoting evidence-based practice with clinicians, there is a strong impetus for university programs to design curricula that will support the development of the knowledge, attitudes, skills and behaviours associated with evidence-based practice. Though occupational therapy curricula in North America are becoming increasingly focused on evidence-based practice, research on students’ attitudes towards evidence-based practice, their perceptions regarding the integration and impact of this content within the curricula, and the impact of the curriculum on their readiness for evidence-based practice is scarce. The present study examined occupational therapy students’ perceptions towards the teaching and assessment of evidence-based practice within a professional master’s curriculum and their self-efficacy for evidence-based practice.

**Methods:**

The study used a mixed methods explanatory sequential design. The quantitative phase included a cross-sectional questionnaire exploring attitudes towards evidence-based practice, perceptions of the teaching and assessment of evidence-based practice and evidence-based practice self-efficacy for four cohorts of students enrolled in the program and a cohort of new graduates. The questionnaire was followed by a focus group of senior students aimed at further exploring the quantitative findings.

**Results:**

All student cohorts held favourable attitudes towards evidence-based practice; there was no difference across cohorts. There were significant differences with regards to perceptions of the teaching and assessment of evidence-based practice within the curriculum; junior cohorts and students with previous education had less favourable perceptions. Students’ self-efficacy for evidence-based practice was significantly higher across cohorts. Four main themes emerged from the focus group data: (a) Having mixed feelings about the value of evidence-based practice (b) Barriers to the application of evidence-based practice; (c) Opposing worlds and (d) Vital and imperfect role of the curriculum.

**Conclusion:**

This study provides important data to support the design and revision of evidence-based practice curricula within professional rehabilitation programs.

**Electronic supplementary material:**

The online version of this article (doi:10.1186/s12909-017-0895-2) contains supplementary material, which is available to authorized users.

## Background

As an approach to clinical decision-making, evidence-based practice (EBP) is defined as the conscientious, explicit, and judicious use of current best evidence in making decisions about the care of individual clients [1]. EBP entails a thoughtful combination of research evidence with clinical expertise, and patient choice, in order to make clinical decisions [1]. EBP has become an integral, guiding framework for occupational therapy (OT) practice [2, 3]. A 2009 position statement on EBP by the Canadian Association of Occupational Therapists (CAOT) urged clinicians to embrace and apply EBP [4].

Though there is a growing body of available research to help guide clinical decisions, utilization of research findings in occupational therapy practice is lacking [5–8]. A number of individual and organizational factors have been associated with poor uptake of EBP. Individual determinants include lack of time to access, read and appraise the literature, limited confidence in applying the principles of EBP, lack of formal education in the principles of EBP, poor research-related knowledge and skills and negative perceptions about the usefulness of research findings in clinical practice [9–12]. A scoping review of the factors that support EBP in occupational therapy practice found that academic degree and post-professional training were the strongest predictors of self-reported use of research evidence [13].

Involvement in research, student supervision, mentoring practices, presence of researchers, advanced practice leaders and librarians on site, university affiliation, collaborations with peers and members of the interdisciplinary team and reflective practice were all associated with greater use of evidence in practice [13].

Organizational determinants and context of healthcare organizations can also influence the use of new knowledge in practice. For example, leadership style, organizational culture, social capital, availability of resources, staffing, time and space, are among the main factors that influence uptake of EBP [14, 15]. The research on organizational determinants of EBP in rehabilitation is scarce, with the few studies conducted in occupational therapy suggesting that systems-level changes and shifts in the organization’s paradigm can reduce barriers and promote a culture of EBP [10, 16–21]. Thomas and Law’s [13] review suggests that transformative leadership styles and employers that embrace and support reflective practice, collaborative learning opportunities and student supervision, contribute towards a climate that supports EBP.

### The role of educational programs in promoting EBP competencies

Studies on the impact of professional education on EBP knowledge, attitudes, skills, behaviours and confidence have produced variable results. A small study on final year OT students (*n* = 86) in Ireland, found that learners understand what EBP entails and are willing to use this approach to inform practice [22]. A qualitative study in the US showed that graduates generally hold positive attitudes toward EBP, view it as patient-centered, as embodying best practices, and as holding promise for the future of the profession [23]. A retrospective cohort study used a survey to explore master’s entry-level (MEL) and bachelor entry-level (BEL) graduates’ reasons for seeking and using information in their practice, the type of information they need most, how they search for it and how the information is used in clinical decision-making. The MEL graduates were more likely (28% vs. 15% for BEL) to use academic health sciences libraries to inform clinical decision-making, found books (56%) and journal articles (63%) to be more useful compared to 43 and 32% for the BEL graduates. MEL graduates were less likely to ask colleagues for information (38%) than their BEL counterparts (79%) and they reported higher success in analyzing and applying scientific findings in practice (40% of BEL vs. 74% of MEL) [24].

A systematic review [25] of eight prospective studies (medical students, *n* = 5 programs; nursing, *n* = 1 program; physical therapy (PT), *n* = 1 program; combined OT/PT, *n* = 1 program) examining the magnitude of change in EBP outcomes following EBP training [25] found sizeable improvements in self-reported EBP knowledge (effect sizes ranged from 0.33 to 5.42) and confidence (effect sizes ranged from 0.89 to 3.03). In comparison, training was associated with a small change in students’ attitudes toward EBP (effect sizes ranged from 0.075 to 0.57). The impact of EBP training on EBP behaviours measured by self-reported use of EBP was found to be widely inconsistent across studies with effect sizes ranging from negligible (0.031) to very large (1.34), a finding that exemplifies the recognized variability in the literature [25]. A small study of 34 senior OT students found declines in confidence (3%) and perceived relevance of research for practice (13%) one year post-graduation [24]. A prospective longitudinal study [26] used a survey to measure changes in self-reported EBP knowledge (understanding of research related terminology), confidence, attitudes and behaviours in two cohorts (*n* = 29 in 2008 and *n* = 76 in 2009) of entry-level PT students, who were transitioning into the workforce. While changes in EBP domains were not significant, the results suggest a decline in self-reported use and perceived relevance of EBP despite increases in confidence and knowledge during the first two years in the work force. This discrepancy highlights the importance of extending EBP training beyond the academic environment to the workplace [26].

If new clinicians are to embrace and effectively incorporate the principles of EBP, they will need to develop the knowledge, skills and attitudes for integrating scientific findings into practice early in their careers and, ideally, during their formal academic training. To this end, the CAOT and its accrediting council expect all occupational therapy programs to design and implement curricula that will promote entry-level competencies in EBP. However, the EBP knowledge, skills and attitudes required at different levels of occupational therapy education, the trajectory of development of EBP competencies throughout the course of formal education and the most effective strategies for implementing EBP in occupational therapy curricula have yet to be identified [27]. Moreover, there is currently scant literature exploring 1) students’ attitudes towards EBP and whether attitudes change as students progress through a program of study and 2) students’ views and experiences of the EBP curriculum and 3) whether the programs are adequately preparing them for their future role as scholarly practitioners. The objective of this study was to examine students’ perceptions of the teaching and assessment of EBP within an occupational therapy professional master’s curriculum.

## Methods

### Design

The study used a mixed-methods sequential design [28]. The quantitative phase consisted of a cross sectional survey of students’ attitudes towards EBP, their perceptions of the impact of the EBP curriculum and their self-efficacy for EBP. The qualitative phase consisted of a focus group with seven senior occupational therapy students designed to explore the survey data in greater depth. Each phase is described in detail below. Ethics approval was obtained from the McGill University Institutional Review Board.

### Participants

Participants were students in a four and one half year MEL professional occupational therapy program from a research-intensive university in Canada and a group of new graduates (one year into practice) from the same university. The first cohort of students (total number of students in the cohort = 72) represented Year 1 (U1) and Year 2 (U2) participants. This group had preliminary exposure to EBP concepts, with basic instruction on how to search the literature and how to critically appraise the research evidence. The second cohort included (total number of students in the cohort = 50) participants in the middle stages of their occupational therapy education known as the “qualifying year” (QY). This cohort (hereafter called Y3) consisted of students either in their third year of the occupational therapy program, or students with a previous undergraduate degree in a discipline other than occupational therapy and were admitted to the third year of the program. All Y3 students must successfully complete their third-year coursework to be admitted into the master’s degree program. Students in this cohort had explicit teaching on the goals and value of EBP and the 5 steps of the EBP process. They also had multiple in class opportunities to implement the EBP process using clinical case scenarios, case based discussions, workshops, and course assignments. The third cohort was comprised of 54 students in their first year of the master’s program (M1). These students had completed three of their four clinical placements and had experienced a curriculum rich with EBP theory and concepts. During the final semester of the professional master’s program students complete a group research project; while working on the project students are given the opportunity to integrate their EBP skills and knowledge with the guidance of clinical and academic supervisors. The final cohort (*n* = 13) included new graduates who had completed the program (M2) between six and 12 months prior to the study, and who had begun their clinical practice. All data were collected during the 2013–2014 academic year. These groups represented different levels of exposure to EBP and clinical (fieldwork) experience (Table 1).

**Table 1:** Description of the five occupational therapy cohorts and their respective exposure to EBP within [masked] University’s School of Physical and Occupational Therapy

Cohort	N	Description	Content	Exposure to EBP
Year 1	42	First year students, primarily with no previous university experience.	Two OT specific courses, remainder of coursework dedicated to physical sciences (e.g., anatomy, physiology).	Students are taught how to perform literature searches using common databases.
Year 2	35	Second year students.	Primarily complementary courses, including human and physical sciences, languages, and research methods.	Basic concepts and rationale for EBP Students are introduced to critical appraisal. EBP concepts introduced in research methods course.
Year 3	76	Two subgroups: U3 – past U1/U2 students. At the end of this year students receive an undergraduate degree in Rehabilitation Sciences (non-practicing). QY - students admitted to the qualifying year with a previous undergraduate degree (or higher). Both groups form one class and receive the same teaching content.	All courses are OT specific.	Explicit teaching of EBP rational and steps of application to clinical scenarios, workshops on conducting appropriate literature searches. • Foundations of EBP: • EBP steps/process • Library workshop on creating a PICO and searching the literature • 3 courses covering concepts of EBP and searching the literature • 1 course covering concepts and process of EBP and integration of evidence EBP guidelines, asking and answering a clinical question, searching to find the answer to a PICO, critical appraisal, classifying evidence on the effectiveness of interventions according to specific guidelines, synthesizing research information for clinical applicability, research design Application of EBP is expected in assignments and exams.
M1	54	Summer semester - clinical placements 1 and 2 (7 weeks each) Fall semester – coursework. Winter semester - clinical placement 3 (7 weeks) Spring semester - coursework Summer semester – group research project	All courses are OT specific.	EBP teaching heavily integrated in lectures and coursework. EBP skills and knowledge developed and refined with clinical application in placements. Research project integrates concepts
M2 (New graduates)	65	Clinical placement 4 (8 weeks) November 2013 graduates, recruited as new clinicians.	Clinical placements.	Application of EBP

### Quantitative phase

The instrument consisted of a questionnaire designed on the basis of the literature on teaching and assessment of EBP and EBP within occupational therapy practice [29–32]. It was developed by the authors and reviewed by a panel of ten professors and researchers in the physical and occupational therapy programs at the university where the research took place. Professors were selected based on their knowledge of the EBP curriculum (e.g., members of the curriculum committee, program director) and their expertise in survey design (e.g., measurement experts). The questionnaire was revised based on the expert feedback and pilot-tested with eight physical therapy students. Physical therapy students in this interprofessional rehabilitation school followed a very similar EBP curriculum and were at the same academic levels as the potential study participants. They were ideally positioned to provide feedback on the questionnaire content, clarity and length. Additional changes were made to improve clarity and content. The final version of the questionnaire was comprised of five sections containing a total of 69 items. (The questionnaire can be found in the Additional file 1). Questions in sections 1–3 were scored on a 7-point Likert scale from ‘strongly disagree’ to ‘strongly agree’.

- **Section 1**: Questions (*n* = 13 items) targeted students’ attitudes towards EBP, including their perceptions of the value of EBP in OT practice.
- **Section 2**: Questions (*n* = 29 items) targeted students’ perceptions of the teaching and assessment of EBP in the curriculum. Specifically, questions targeted perceptions of the emphasis of EBP in the program, the teaching strategies used, the types of assessment methods and the value of having clinician lecturers.
- **Section 3**. This section (*n* = 11 items) explored students’ experience with EBP in their fieldwork placements. The questions focused on the opportunities for EBP in the clinical setting and preceptors’ roles in promoting EBP.
- **Section 4**: This section assessed students’ self-efficacy in EBP, measured by the evidence-based practice confidence (EPIC) scale. The EPIC contains 11 items that target the 5-step EBP process (ask, appraise, acquire, apply, assess). Participants rate their level of confidence on an 11-point scale (0% confidence to 100% confidence) [33]. Item-level scores can be averaged to obtain a total score ranging from 0 to 100 percentage points. The scale has excellent internal consistency (0.89; 95% confidence interval 0.86 to 0.91) and test-retest reliability (0.89; 95% confidence interval 0.85 to 0.91) [34].
- **Section 5**. This final section consisted of questions on demographic variables such as age, academic year, previous degrees held, previous research experience, and grade point average.

### Qualitative phase

A 90-min focus group led by two graduate-level research assistants was conducted with seven students from the most senior cohort in the program (master’s year 1). Having completed three years in the program and three fieldwork rotations totalling over 700 h, this group had a broader perspective of the teaching and assessment of EBP within the curriculum. These senior students had a historical perspective of the curriculum and could potentially provide rich data to shed light on the questionnaire results as well as provide suggestions for improving the EBP content across the curriculum. Focus group questions were developed following a preliminary analysis of the questionnaire results and were designed to gain a deeper understanding of the quantitative findings (The focus group interview protocol can be found in the Additional file 2).

### Recruitment and data collection

- **Questionnaire**. An email explaining the purpose of the study and asking for permission to contact students was sent to the program director. Once permission was granted, the research team contacted each cohort's course instructor explaining the study and asking for permission to visit students at the beginning of one of their classes. Students interested in participating in the study were asked to complete the questionnaire and place it in a sealed and masked envelope at the front of the class. A research assistant collected the questionnaires. The new graduates were recruited via an email sent by the program director inviting them to complete an online version of the questionnaire (Fluid Surveys). This group was given two weeks to complete the questionnaire with a reminder after one week.
- **Focus group**. Students from the M1 cohort who completed the questionnaire were invited by email to participate in the focus group. Two graduate level research assistants led the 90-min focus group. The focus group discussion was audio recorded and transcribed verbatim.

### Analysis

#### Quantitative

- *Questionnaire data sections 1 and 2*: Data were analysed using SPSS version 22. Due to a low response rate from each group, years 1 and 2 (Y1/Y2) data were combined and analysed as one cohort. The two cohorts have comparable exposure to EBP content within the program. All questionnaire items missing more than three responses were removed. This resulted in a total of 10 questions for Section 1 and 24 questions for Section 2. Mean imputation [35, 36] was used to analyse items with three missing responses or less. A Cronbach’s alpha was obtained for each section. A mean score was calculated for each section; a higher mean score indicated more favourable attitudes towards EBP. Descriptive statistics (means and standard deviations) were obtained for each section and cohort. A one-way analysis of variance (ANOVA) and post hoc t-tests (Tukey’s Honestly Significant Difference) were conducted on each section of the questionnaire to examine differences between cohorts.
- *Questionnaire data section 3*: Perceptions regarding EBP in fieldwork are not reported in this paper, as a comparison across all cohorts was not possible; only M1 and M2 students had clinical experience and could complete this section.
- *Questionnaire data section 4*: Descriptive statistics and a one-way ANOVA with post hoc tests were also obtained for the EBP self-efficacy scores.

#### Qualitative

The focus group discussion was recorded, transcribed verbatim and analysed using a descriptive thematic analysis approach [37]. Two members of the research team independently coded the transcript. They identified units of meaning and developed preliminary categories. The research team including the senior researcher (AT) discussed the categories and emerging themes and resolved disagreement through discussion.

## Results

Of the 207 eligible students 115 (56%) completed the questionnaire: 17 Y1/Y2 (22%), 50 Y3 (66%) and 48 M1 (89%) students. Thirteen of the 65 new graduates (22%) completed the questionnaire. Demographic data are provided in Table 2.

**Table 2:** Demographic information of participants

Cohort	Class size	*n*	Response rate (%)	Previous education		Research experience		GPA	
				Yes	No	Yes	No	Above 3.7	Below 3.7
U1/U2	77	17	22	0	17	1	16	4	13
U3/QY	76	50	66	23	27	14	36	12	38
M1	54	48	89	24	24	16	32	35	13
New Grads	65	13	22	6	7	6	7	12	13

- **Section 1**. Cronbach’s alpha for section 1 was .71 (Table 3). The overall mean score for attitudes towards EBP for the Y1/Y2 cohort was 5.29, SD = 0.61, 5.40, *SD* = 0.56 for the Y3 and 5.35, *SD* = 0.69 for the M1 cohort. The new graduates’ mean score on attitudes towards EBP was 5.46, *SD* = 0.42. The ANOVA indicated no significant differences in attitudes across the groups, *F*(3,124) = .253, *p* = .86.

**Table 3:** Mean scores on the EBP survey per cohort

		Cohort				Cronbach’s Alpha
Section	N (items)	U1/U2	U3/QY	M1	New Grads	
Section 1: Attitudes^a^	10	5.29 (0.61)	5.40 (0.56)	5.35 (0.69)	5.46 (0.42)	0.70
Section 2: Curriculum^a^	24	4.42 (1.12)	5.04 (0.65)	5.12 (0.65)	5.53 (0.56)	0.90
Section 4: EPIC^b^	11	52.46 (23.84)	61.44 (14.69)	68.33 (8.68)	78.46 (9.80)	0.91

The table presents mean scores on the EBP Survey per cohort, with standard deviations in parentheses

^a^Where 7 represents strongly agree and 1 represents strongly disagree

^b^Where a higher percentage is equal to higher self-efficacy
- **Section 2**. Cronbach’s alpha for Section 2 regarding *students’ perceptions of the teaching and assessment of EBP in the curriculum* was .90 (Table 3). The overall mean score for Y1/Y2 was 4.42, *SD* = 1.12, 5.04, *SD* = 0.65 for the Y3 students, 5.12, *SD* = 0.65 for the M1 cohort, and 5.53, SD = 0.56 for the new graduates. There were significant differences amongst cohorts *F*(3,124) = 6.823, *p* < .001. The Y1/Y2 students’ perceptions of the teaching and assessment of EBP in the curriculum were significantly less favourable than those of the Y3 cohort (*p* = 0.01), the M1 students (*p* < .001), as well as the new graduates (*p* < .001). There were no significant differences between Y3 and M1 (*p*=. 940), Y3 and new graduates (*p* = .113), or between M1 and new graduates (*p* = .241).
- **Section 4**. EBP self-efficacy. There were significant differences among participants’ confidence in their ability to use EBP, *F*(3,124) = 10.47, *p <* .001). The M1 cohort (*M* = 68.33, *SD* = 8.68) and the new graduates (*M* = 78.46, *SD* = 9.80) had significantly higher EBP self-efficacy than the Y1/Y2 students (M = 52.46, SD = 23.84, *p <* .001). The M2 cohort also had significantly higher self-efficacy than the Y3 cohort (*M* = 61.44, *SD* = 14.69, *p <* .001). There was no significant difference in self-efficacy between the M1 and Y3 cohort (*p* = .08).

### Qualitative results

Analysis of the focus group data resulted in four major themes.

- **Theme 1**: **Having mixed feelings about the value of EBP**. This theme highlighted students’ perceptions of the impact of EBP on the profession, the value that students place on EBP for everyday occupational therapy practice and the importance of using various sources of evidence. Four categories were nested within this theme: (a) other avenues for obtaining evidence, (b) affords credibility and an identity to the profession, (c) doing what’s best, and (d) nonconforming.
  - *Other avenues for obtaining evidence*. Students generally viewed EBP as positive but reported that research literature may not be the only source of information for an evidence-informed approach to decision-making. For example, one student noted, “[it’s] not just searching the literature, but also conferences and training, then that’s just as good.”
  - *Affords credibility and an identity to the profession.* Participants emphasized that EBP gives credibility to the profession and helps occupational therapists form and strengthen their identity as a profession. One student said, “I think supporting our profession with [EBP] is really important, it gives us credibility.”
  - *Doing what’s best.* Students viewed EBP as beneficial for guiding client-centred best practice and focusing on clients’ needs and wishes. One participant stated, “OT is such a grey profession that it’s good to have EBP to know what’s the best thing to do for clients and confirm our role.”
  - *Nonconforming to organizational pressures.* In addition to the perceived value of EBP and recognition of its many benefits for occupational therapy practice, participants reported that being evidence-based at times seemed like nonconforming to institutional practices. One student said, “You proactively look at the evidence and choose what to do based on that rather than being told it’s like that in this institution so do that.”
- **Theme 2**: **Barriers to the application of EBP**. This theme revealed students’ perceptions of the feasibility of using EBP and the potential barriers to its implementation in daily practice. There were four nested categories: (a) dealing with limited time, (b) EBP is challenging to integrate, (c) appropriate and sufficient resources are required to implement EBP, and (d) lacking clarity on how to apply evidence in occupational therapy.
  - *Dealing with limited time*. Participants reported that there is a lack of time in the clinical environment to apply EBP in comparison to the school environment. For example, one student expressed, “I don’t know if I’ll …have enough time. Because I feel like doing research is very time consuming.”
  - *EBP is challenging to integrate*. Students indicated that EBP could be difficult to integrate because of competing demands on clinicians’ time as one student said “there’s also other things to do, like charting”.
  - *Appropriate and sufficient resources are required to implement EBP*. Students mentioned that there is also a lack of resources in the clinical environment in comparison to the resources at their disposal in the classroom setting. One student explained, “Right now we have access to everything but once we start in the workforce…what we have access to is so limited that it doesn’t give you as much opportunity to be evidence-based.” Another student commented on how clinicians have access to more resources because of their supervisory activities: “supervisors often only have access to EBP resources through us.”
  - *Lacking clarity on how to apply evidence in occupational therapy*. Participants also noted that the application of evidence in occupational therapy is not always clear. For occupational therapists, treatments may require alterations and adaptions to fit client needs as exemplified by one student who said, “It’s not so clear cut. Like you really have to look at each client and what works with each client.”
- **Theme 3**: **Opposing worlds**. This theme highlighted contrasts between using EBP in an ideal world and using EBP in everyday practice. The theme captured several dichotomies within EBP as represented by the following categories: (a) contrast between school and clinical practice, (b) reality vs. idealism, (c) balance between clinical experience and evidence, (d) tension between client’s wishes and EBP, (e) unease with Bachelor’s level training vs. Master’s level training, and (f) anticipation of clinical reality: occupational therapists vs. evidence-based practitioners.
  - *Contrast between school and clinical practice.* Participants reported noticing a contrast between the EBP-friendly, resource rich academic environment, and the realities of clinical practice. With its plethora of resources and opportunities, the academic setting lends itself easily to EBP; in contrast, the clinical environment presents several challenges including limited time, scarce resources, competing client wishes, and numerous clinical responsibilities, all of which contribute towards making EBP more difficult. One student shared: “What you see in your textbook doesn’t always apply. […] It’s kind of embarrassing too. Cause [the supervisors are] like no it’s not gonna work, like don’t even think about it, or it’s not feasible, but you were taught this in school ‘cause it’s ideal but not realistic.”
  - *Reality* vs. *idealism.* There was also a clear dichotomy expressed by participants regarding reality vs. idealism as it relates to the assessments and interventions learned in school, and those that are feasible within the clinical environment. One student said, “There’s this really big clash when we go to clinical placements and the supervisor says you’re not in school, this is reality.”
  - *Balance between clinical experience and evidence*. Participants perceived a difference between clinical skills and the use of evidence in practice: “I think my priority will be being confident in my own skills as a clinician before being confident in my evidence-based practice.”
  - *Tension between client’s wishes and EBP.* Participants also expressed a tension between clients’ wishes and EBP. They shared that what the client wants is not always congruent with EBP. This misalignment forced them to return to the literature or resort to other forms of treatments. As one participant remarked: “It’s also what your client is willing to do. What it says in the literature versus your client’s willingness to participate or actually engage in what you think might be the best way to go about it may not actually go hand in hand.”
  - *Unease with bachelor’s level training* vs. *master’s level.* Students expressed concerns with the fact that most current supervisors hold Bachelor’s entry-level degrees, with minimal formal education in EBP, whereas the “new generation” of clinicians hold professional master’s degrees with extensive preparation in EBP. Although this “new generation” may be attempting to apply EBP from the begining of their clinical practice, they do not have the requisite clinical experience to ensure optimal integration of EBP principles. One student commented, “I think there’s maybe like a clash…most of my supervisors…went through the three-year program [B.Sc.] instead of the four-year and a half [M.Sc.]…we’re finishing with a master’s and they’re finishing with a bachelor’s. And you don’t want to come up and be like I know this better because I have like one year and a half more than you. … She had like eight years experience, and that counts too.”
  - *Anticipation of clinical reality: occupational therapists* vs. *evidence-based practitioners.* There was also a perceived distinction between the occupational therapist and the evidence-based practitioner. Participants conveyed that being a good occupational therapist is not necessarily synonymous with being an evidence-based practitioner, emphasizing the importance of experience as a contributor to good clinical practice. As one student noted: [EBP] won’t be my priority. At first I’ll just learn what I have to do. And then I’ll try and incorporate more evidence and see what’s best. But I think at first you need to understand where you’re working and how it’s gonna work.”
- **Theme 4**: **Vital and imperfect role of the curriculum**. This theme captured students’ perceptions of the curriculum and its impact on the acquisition of EBP knowledge, skills and attitudes. The theme underscores the importance of education in promoting EBP competencies but also highlights its imperfections. The theme comprised six categories: (a) differing backgrounds; (b) academic strategies and resources for learning EBP; (c) readiness and preparedness; (d) academic support for EBP in the clinical environment; (e) small class size as a facilitator/more direct time; and (f) impact of fieldwork.
  - *Differing backgrounds.* Participants expressed that differences in the level of EBP knowledge and skills associated with some students' academic backgrounds created inequalities in terms of learning. This was particularly true for those admitted to the program with a previous undergraduate degree. One participant suggested that “[all students] should be “on equal playing ground when they enter the program” and another said, “EBP should be taught from the very beginning of the curriculum. I think we should be more pushed into EBP from the start.”
  - *Academic strategies and resources for learning EBP.* Participants also reflected upon available academic strategies and resources for learning EBP, identifying sessions with the librarian, case-review-workshops, and case-based-assignments as helpful. The importance of having professors readily available when they needed assistance with EBP-related material was discussed, and highlighted by one student who said, “But if they have hours, then I can just swing by and ask them the question.”
  - *Feeling ready and prepared.* Students spoke about readiness and preparedness for EBP when they begin fieldwork, based on resources provided by the program. Some felt that having professors or guest clinicians share their clinical experience might shed light on the real life application of EBP. One student stated, “Encouraging them [clinicians or professors] to talk about their experience of actually applying it [EBP] and the difficulties that they come across, or… like the reality of it would make me feel more prepared to be able to apply EBP as a clinician.”
  - *Accessing academic support for EBP in the clinical environment.* Students reported a need for academic support for EBP in the clinical environment and felt that it is the school’s responsibility to ensure clinical preceptors are aware of the importance of EBP. For example, one student suggested that “[the program should] encourage all supervisors to provide us with time to … research something to do.”
  - *Small class size as a facilitator/more direct time.* Participants indicated that small class sizes and direct time with professors were factors that could support the development of competence in EBP*.* They expressed that in smaller classroom settings, they felt more comfortable approaching professors with questions regarding EBP, and class assignments tended to be more oriented towards encouraging the use of EBP, as one student expressed “[being in] smaller elective classes, EBP was integrated a lot more, and it was more of a comfortable environment to ask questions.”
  - *Impact of fieldwork.* Students identified the clinical fieldwork environment as the place where they learn how EBP is applied in practice and how to overcome barriers. In fact, students expressed that in fieldwork they learn faster and how best to implement EBP. Fieldwork provided students with an appreciation of the importance of best practices, as one student expressed, “you learn faster in clinical placement because” and “[clinical placement] made [EBP] really concrete […] to actually apply it and try what was done.” Overall, participants expressed a heightened awareness of EBP, including its barriers, facilitators, and uses, within the context of their fieldwork experiences.

## Discussion

Though the importance of professional education for promoting EBP and shaping EBP competencies (knowledge, attitudes, skills) has been well documented in the literature [13, 28, 30, 31], there has been little attention paid to students’ perceptions of the teaching and assessment of EBP and their confidence in their ability to apply EBP. We expanded upon the work of others (e.g., [22]) to explore students’ perceptions of the value of EBP and of the teaching and assessment of EBP within an occupational therapy professional master’s curriculum.

The findings suggest that overall, students’ attitudes towards EBP were moderately positive, which is consistent with the findings from other studies [18, 22, 23] though there is room for improvement. Students appear to value EBP particularly as it enhances the profession’s credibility. However, attitudes towards EBP were not significantly different across the cohorts. This finding was unexpected given the increasing emphasis and opportunity to apply EBP as students progress through the curriculum. Haas and colleagues suggest that with increased exposure to EBP and as students experience barriers to implementing EBP in practice, the “lustre” may fade [38]. The findings from our focus groups revealed that while students value EBP and believe that it adds credibility to the profession, the challenges experienced in implementing EBP in their fieldwork placements had a less than favourable impact on their perceptions of EBP. Considering the sub-optimal implementation of EBP by busy clinicians [7, 12, 39], it is not surprising that students are exposed to environments where there is limited uptake of best practices. Another possible explanation for the lack of significant differences across cohorts is that although the exposure to EBP increases from year to year (i.e., students are instructed on its importance for clinical practice and value for the profession and progressively learn how to apply EBP), the curriculum may not be designed in a way to specifically target attitudes towards EBP. In other words, EBP exposure may be progressively more focused on the “doing” rather than on the “valuing and embracing”.

To our knowledge, there is no other research on students’ perceptions and experiences of the teaching and assessment of EBP within occupational therapy curricula. However, given the incremental exposure to, and practice with EBP across the four and one half years of the curriculum, we anticipated that students’ perceptions would be increasingly more favourable. The focus group data shed some light on a possible reason for this. The senior students who had completed almost 4 years of the program expressed that professors and guest speakers need to use more concrete examples to demonstrate the clinical applications of EBP. Moreover, given that many professors are ‘removed” from clinical practice, students suggested that more clinicians should be involved in the curriculum and at different points in time. Students reported feeling frustrated with the lack of consistency across years with respect to EBP content, methods used to teach EBP and clinical applications. This finding highlights the vital role that curriculum committees have in updating, reviewing and revising core EBP content in a dynamic and ongoing manner.

Our results revealed significant differences between novice (Y1/Y2) and more senior students’ (M1/M2) perceptions of the nature and amount of teaching and assessment of EBP in the curriculum. More specifically, students in the senior cohorts (i.e., M1/M2) have more favourable perceptions than those in the junior cohorts (i.e., YI/Y2). These findings suggest that increased exposure and opportunity to apply EBP may lead to more positive perceptions of the curriculum, a finding corroborated by other research on the impact of integrated and longitudinal EBP education in medical programs [32, 40]. Interestingly, the focus group data suggest that for the few students with previous degrees, there was a comparison point; they could use past academic experiences of EBP, including teaching and assessment methods, to compare and contrast with the present curriculum. They were therefore in a position to be more critical and to identify gaps in the curriculum. In such situations, programs may consider ‘mixing” students with different backgrounds for group assignments and classroom activities so that those with less academic exposure to EBP can benefit from the experience of students with previous academic degrees.

Students made several suggestions for improving instruction of EBP in the curriculum. Not surprisingly, students suggested having smaller class sizes and more “real-world” examples. This is consistent with much of the literature suggesting that authentic and situated learning experiences provide students with clinical scenarios that are closest to those that they will encounter in their practice [27, 32]. A systematic review of the teaching of EBP has reported that improved knowledge, skills, and attitudes resulted from multifaceted and clinically integrated learning opportunities, such as using real clinical issues, small group discussions, and journal clubs [32].

Results revealed significant differences between cohorts in EBP self-efficacy with advancing academic level. The senior cohort (M1) had 700 h of fieldwork experience; the highest number of contact hours on EBP in the curriculum. This finding is consistent with other studies having found a relationship between amount of exposure to EBP and self-efficacy [41, 42]. Surprisingly, there was no significant difference between Y1/Y2 and Y3. Students in the two first years of the program have little exposure to research and EBP, whereas the Y3 cohort has extensive exposure to and practice with EBP in eight different courses. One possible explanation for this finding is that students are still acquiring the requisite knowledge and skills associated with EBP and that these have yet to translate into greater confidence in their ability to apply EBP. Given that the Y3 cohort had not yet begun fieldwork, this finding cannot be attributed to the barriers encountered in the clinical setting.

## Limitations

There are two major limitations in this study. First, the cross-sectional nature of the research does not allow us to capture change in perceptions over time. Second, we could not differentiate between year 1 and year 2 students’ attitudes and self-efficacy as the numbers of participants were too small and the groups had to be collapsed.

## Conclusions

Professional education is believed to play an important role in the development of positive attitudes towards EBP skills and learners’ ability to apply EBP. As key stakeholders, students offer unique perspectives on the strengths and challenges of their educational preparation as well as on their readiness to embrace and apply their roles as scholarly practitioners. Findings from this research provide insights into students’ perceptions of the teaching and assessment of EBP that can be used for purposes of curriculum revision. This mixed methods study adds to the growing literature base on the effectiveness and impact of various models of instruction on EBP. The findings suggest that it is essential to have a well-developed EBP curriculum in order to ensure that graduates are confident and competent evidence-based practitioners. Students favor small class sizes and appreciate having increased access to instructors. Students seek more one-on-one time (student-instructor) as this contributes to greater learning on how to apply EBP and a sense of readiness and confidence. Students believe that *application* of EBP in the clinical context is of utmost importance and that the academic program should design opportunities for this to happen as often as possible. Students acknowledge that EBP is a foundation of the profession and as such, it needs to be emphasized in both the school and clinical environments. This is in line with an emerging trend towards integrated knowledge translation and the scholarship of practice [43–45]. Clinician - academic collaborations have been discussed extensively in the scholarship of practice literature [45, 46]. *Scholarship of practice* is a collaborative model in which theory, research, and practice are interwoven and whereby collaboration between scholars and practitioners foster knowledge dissemination and use [45]. This may be an equally promising model for involving clinicians in both the design and delivery of EBP curricula within university programs and developing partnerships with clinical stakeholders to explore the ways in which the clinical environment can best support the enactment of EBP during students’ fieldwork education.

Future research could include longitudinal studies following one cohort throughout the program to better understand the changes and transitions in learners’ attitudes and confidence in their ability to apply EBP. Additionally, the findings from the fieldwork section of the questionnaire were not reported in this paper, as we could not compare these results across all cohorts. Given the significance of fieldwork in shaping attitudes and self-efficacy as identified in the focus group, future research could explore the impact of fieldwork placements in promoting EBP competencies. A large number of barriers to EBP were identified in the focus group, comprised of students who may soon be facing these barriers as clinicians. It would be interesting to interview new graduates once they have transitioned to clinical practice to identify if they were able to overcome the barriers and apply EBP.

## Additional files

Additional file 1: This file contains the final questionnaire with all 5 sections. (DOCX 40 kb)

Additional file 2: This file contains the focus group interview protocol. (DOCX 15 kb)

## Abbreviations

BEL: Bachelor entry-level
CAOT: Canadian association of occupational therapists
EBP: Evidence-based practice
EPIC: Evidence based practice confidence
ES: Effect sizes
MEL: Master’s entry-level
OT: Occupational therapy
PT: Physical therapy
