# Supplementary material for: Students’ attitudes and perceptions of teaching and assessment of evidence-based practice in an occupational therapy professional Master’s curriculum: a mixed methods study
Source: BMC Med Educ. 2017 Mar 27;17:64. doi: 10.1186/s12909-017-0895-2 (PMC5368912; doi:10.1186/s12909-017-0895-2)
Supplement: Supplementary file 1 — This file contains the final questionnaire with all 5 sections. (DOCX 40 kb) [file 12909_2017_895_MOESM1_ESM.docx]

**Final Questionnaire**

**Section 1 is comprised of 13 questions about your opinions regarding EBP. Indicate to what extent you agree or disagree with the following statements. Please circle your response.**

| Please indicate to what extent you agree or disagree with each of the following statements? | | | | | |  |  |  |
| --- | --- | --- | --- | --- | --- | --- | --- | --- |
|  | Strongly Disagree | Disagree | Somewhat Disagree | Neither Agree nor Disagree | Somewhat Agree | Agree | Strongly Agree | I don’t know |
| **1. EBP is considered an essential element of OT practice** | 1 | 2 | 3 | 4 | 5 | 6 | 7 | 0 |
| **2. EBP adds credibility to the profession of OT** | 1 | 2 | 3 | 4 | 5 | 6 | 7 | 0 |
| **3. Using EBP improves the quality of care delivered to clients** | 1 | 2 | 3 | 4 | 5 | 6 | 7 | 0 |
| **4. Using EBP helps therapists stay informed about new OT treatment interventions** | 1 | 2 | 3 | 4 | 5 | 6 | 7 | 0 |
| **5. It is important for OT clinicians to keep up-to-date with research evidence** | 1 | 2 | 3 | 4 | 5 | 6 | 7 | 0 |
| **6. EBP ignores the client’s wishes** | 1 | 2 | 3 | 4 | 5 | 6 | 7 | 0 |
| **7. Research findings are presented in a language that is difficult to understand** | 1 | 2 | 3 | 4 | 5 | 6 | 7 | 0 |
| **Applications to clinical practice are not always outlined in research studies** | 1 | 2 | 3 | 4 | 5 | 6 | 7 | 0 |

|  | Strongly Disagree | Disagree | Somewhat Disagree | Neither Agree nor Disagree | Somewhat Agree | Agree | Strongly Agree | I don’t know |
| --- | --- | --- | --- | --- | --- | --- | --- | --- |
| **EBP is a “cookie-cutter” approach to therapy** | 1 | 2 | 3 | 4 | 5 | 6 | 7 | 0 |
| **EBP disregards clinical experience** | 1 | 2 | 3 | 4 | 5 | 6 | 7 | 0 |
| **Clinical experience is more important than research evidence when making clinical decisions** | 1 | 2 | 3 | 4 | 5 | 6 | 7 | 0 |
| **EBP takes too much time** | 1 | 2 | 3 | 4 | 5 | 6 | 7 | 0 |
| **EBP is only possible when the appropriate resources are available** | 1 | 2 | 3 | 4 | 5 | 6 | 7 | 0 |

**Section 2 is comprised of 29 questions regarding your perceptions of the teaching and assessment of EBP in the OT curriculum at McGill. It is comprised of 3 subsections: 1) General Impressions, 2) EBP teaching, and 3) EBP Evaluation. Indicate to what extent you agree or disagree with the following statements as it relates to your experience in this program only. Please circle your response.**

| Section 2a: Overall Perception of EBP in the Curriculum  Please indicate to what extent you agree or disagree with each of the following statements? | | | | | |  |  |  |
| --- | --- | --- | --- | --- | --- | --- | --- | --- |
|  | Strongly Disagree | Disagree | Somewhat Disagree | Neither Agree nor Disagree | Somewhat Agree | Agree | Strongly Agree | I don’t know |
| **1. EBP is an integral part of the OT curriculum** | 1 | 2 | 3 | 4 | 5 | 6 | 7 | 0 |
| **2. It is the responsibility of the OT program to help me become an evidence-based practitioner** | 1 | 2 | 3 | 4 | 5 | 6 | 7 | 0 |
| **3. EBP is integrated in all of our professional courses** | 1 | 2 | 3 | 4 | 5 | 6 | 7 | 0 |
| **4. This program has provided me with a strong foundation in EBP** | 1 | 2 | 3 | 4 | 5 | 6 | 7 | 0 |
| **5. This program emphasizes the importance of personal judgment when it comes to implementing EBP** | 1 | 2 | 3 | 4 | 5 | 6 | 7 | 0 |
| **6. I feel comfortable asking professors to explain research findings that I do not understand** | 1 | 2 | 3 | 4 | 5 | 6 | 7 | 0 |

|  | Strongly Disagree | Disagree | Somewhat Disagree | Neither Agree nor Disagree | Somewhat Agree | Agree | Strongly Agree | I don’t know |
| --- | --- | --- | --- | --- | --- | --- | --- | --- |
| **7. I feel comfortable asking professors to explain the clinical applications of research evidence** | 1 | 2 | 3 | 4 | 5 | 6 | 7 | 0 |
| **8. My professors present the clinical applications of research evidence** | 1 | 2 | 3 | 4 | 5 | 6 | 7 | 0 |
| **9. My professors are good role models for EBP** | 1 | 2 | 3 | 4 | 5 | 6 | 7 | 0 |
| **10. My professors demonstrate positive attitudes towards EBP in the classroom** | 1 | 2 | 3 | 4 | 5 | 6 | 7 | 0 |
| **11. Guest clinical lecturers incorporate research evidence into their teaching** | 1 | 2 | 3 | 4 | 5 | 6 | 7 | 0 |
| **12. Guest clinical lecturers are good role models for EBP** | 1 | 2 | 3 | 4 | 5 | 6 | 7 | 0 |
| **13. Guest clinical lecturers help me understand how to incorporate evidence into practice in today’s clinical environment** | 1 | 2 | 3 | 4 | 5 | 6 | 7 | 0 |

| Section 2b: Perception of EBP Training in the Curriculum  Please indicate to what extent you agree or disagree with each of the following statements? | | | | | |  |  |  |
| --- | --- | --- | --- | --- | --- | --- | --- | --- |
|  | Strongly Disagree | Disagree | Somewhat Disagree | Neither Agree nor Disagree | Somewhat Agree | Agree | Strongly Agree | I don’t know |
| **1. I have received adequate training in order to formulate an answerable research question in the PICO format** | 1 | 2 | 3 | 4 | 5 | 6 | 7 | 0 |
| **2. I have received adequate instruction in order to search for scientific articles** | 1 | 2 | 3 | 4 | 5 | 6 | 7 | 0 |
| **3. I have received adequate training in order to critically appraise the scientific articles I find** | 1 | 2 | 3 | 4 | 5 | 6 | 7 | 0 |
| **4. I have received adequate training in order to understand the different levels of evidence for treatment effectiveness** | 1 | 2 | 3 | 4 | 5 | 6 | 7 | 0 |
| **5. I have received adequate training to help me understand different kinds of scientific research designs(randomized control trail, cohort study, cross-sectional) in order to implement EBP** | 1 | 2 | 3 | 4 | 5 | 6 | 7 | 0 |

|  | Strongly Disagree | Disagree | Somewhat Disagree | Neither Agree nor Disagree | Somewhat Agree | Agree | Strongly Agree | I don’t know |
| --- | --- | --- | --- | --- | --- | --- | --- | --- |
| **6. I have received adequate training on how to apply the results of various scientific research articles/study designs to clinical case scenarios** | 1 | 2 | 3 | 4 | 5 | 6 | 7 | 0 |
| **7. I need more EBP instruction in order to apply it in practice** | 1 | 2 | 3 | 4 | 5 | 6 | 7 | 0 |
| **8. My assigned course readings help me keep up-to-date with research evidence** | 1 | 2 | 3 | 4 | 5 | 6 | 7 | 0 |
| **9. I would rather learn about interventions from clinicians than from scientific literature** | 1 | 2 | 3 | 4 | 5 | 6 | 7 | 0 |
| **10. Clinical case scenarios help me apply EBP in the classroom** | 1 | 2 | 3 | 4 | 5 | 6 | 7 | 0 |
| **11. My coursework (e.g. assignments, readings, papers, quizzes). helps me to understand** **how I can apply EBP in the clinical context** | 1 | 2 | 3 | 4 | 5 | 6 | 7 | 0 |
| **12. I am comfortable using systematic reviews to gather evidence** | 1 | 2 | 3 | 4 | 5 | 6 | 7 | 0 |

| Section 2c: Perception of EBP Evaluation in the Curriculum  Please indicate to what extent you agree or disagree with each of the following statements? | | | | | |  |  |  |
| --- | --- | --- | --- | --- | --- | --- | --- | --- |
|  | Strongly Disagree | Disagree | Somewhat Disagree | Neither Agree nor Disagree | Somewhat Agree | Agree | Strongly Agree | I don’t know |
| **1. I am encouraged to use research in my class assignments** | 1 | 2 | 3 | 4 | 5 | 6 | 7 | 0 |
| **2. I receive feedback from my professors about the quality of scientific evidence I use in my assignments** | 1 | 2 | 3 | 4 | 5 | 6 | 7 | 0 |
| **3. Exams adequately evaluate my learning of EBP concepts** | 1 | 2 | 3 | 4 | 5 | 6 | 7 | 0 |
| **4. Class assignments (i.e. CBAs, midterm papers, etc) adequately evaluate my learning of EBP concepts** | 1 | 2 | 3 | 4 | 5 | 6 | 7 | 0 |

**Section 3 consists of 11 questions regarding your experience of EBP in your fieldwork placements. If you have not yet taken a practicum (U1-U3/QY), please proceed to Section 4.**

**Complete this section ONLY if you have completed a practical placement or are now working as an OT (M1, M2). Indicate to what extent you agree or disagree with the following statements. Please circle your response.**

| Please indicate to what extent you agree or disagree with each of the following statements? | | | | | |  |  |  |
| --- | --- | --- | --- | --- | --- | --- | --- | --- |
|  | Strongly Disagree | Disagree | Somewhat Disagree | Neither Agree nor Disagree | Somewhat Agree | Agree | Strongly Agree | I don’t know |
| **1. During my fieldwork experiences I had opportunities to apply the EBP knowledge and skills acquired from my academic program** | 1 | 2 | 3 | 4 | 5 | 6 | 7 | 0 |
| **2. The settings in which I had my fieldwork experience promoted EBP** | 1 | 2 | 3 | 4 | 5 | 6 | 7 | 0 |
| **3. I did not have enough time to use EBP during my fieldwork experiences** | 1 | 2 | 3 | 4 | 5 | 6 | 7 | 0 |
| **4. My clinical educators were good role models for using EBP** | 1 | 2 | 3 | 4 | 5 | 6 | 7 | 0 |
| **5. During my fieldwork experiences, I had adequate time to search for evidence** | 1 | 2 | 3 | 4 | 5 | 6 | 7 | 0 |
| **6. In my fieldwork experiences, I was encouraged to implement EBP** | 1 | 2 | 3 | 4 | 5 | 6 | 7 | 0 |
|  | Strongly Disagree | Disagree | Somewhat Disagree | Neither Agree nor Disagree | Somewhat Agree | Agree | Strongly Agree | I don’t know |
| **7. During my fieldwork experiences, I saw the value of using EBP to guide clinical decisions** | 1 | 2 | 3 | 4 | 5 | 6 | 7 | 0 |
| **During my fieldwork experiences, I had access to the required resources to search for evidence** | 1 | 2 | 3 | 4 | 5 | 6 | 7 | 0 |
| **I was sufficiently prepared to implement EBP when I began my fieldwork experiences** | 1 | 2 | 3 | 4 | 5 | 6 | 7 | 0 |
| **During my fieldwork experiences, I felt comfortable discussing the application of EBP with my supervisor** | 1 | 2 | 3 | 4 | 5 | 6 | 7 | 0 |
| **My supervisor provided feedback on my EBP skills** | 1 | 2 | 3 | 4 | 5 | 6 | 7 | 0 |

**Section 4: Evidence-based practice confidence (EPIC) scale and Evidence Based Practice (EBP) self-efficacy scale (**Salbach et al, 2010; Salbach et al, 2013**). This section consists of 11 questions about your confidence in applying the various steps of the EBP process.**

For each of the following activities, please indicate how **confident** you are in your **current** level of ability by choosing the corresponding number on the following rating scale:

| ○ **0%** | ○ **10** | ○ **20** | ○ **30** | ○ **40** | ○ **50** | ○ **60** | ○ **70** | ○ **80** | ○ **90** | ○ **100%** | |
| --- | --- | --- | --- | --- | --- | --- | --- | --- | --- | --- | --- |
| **No Confidence** | |  |  |  |  |  | **Completely Confident** | | | |  |

How **confident** are you in your ability to:

|  | … identify a gap in your knowledge related to a patient or client situation (e.g., history, assessment, treatment)? | ○ **0%** | ○ **10** | ○ **20** | ○ **30** | ○ **40** | ○ **50** | ○ **60** | ○ **70** | ○ **80** | ○ **90** | ○ **100%** |
| --- | --- | --- | --- | --- | --- | --- | --- | --- | --- | --- | --- | --- |
|  | … formulate a question to guide a literature search based on a gap in your knowledge? | ○ **0%** | ○ **10** | ○ **20** | ○ **30** | ○ **40** | ○ **50** | ○ **60** | ○ **70** | ○ **80** | ○ **90** | ○ **100%** |
|  | … effectively conduct an online literature search to address the question? | ○ **0%** | ○ **10** | ○ **20** | ○ **30** | ○ **40** | ○ **50** | ○ **60** | ○ **70** | ○ **80** | ○ **90** | ○ **100%** |
|  | … critically appraise the strengths and weaknesses of study methods (e.g., appropriateness of study design, recruitment, data collection and analysis)? | ○ **0%** | ○ **10** | ○ **20** | ○ **30** | ○ **40** | ○ **50** | ○ **60** | ○ **70** | ○ **80** | ○ **90** | ○ **100%** |
|  | … critically appraise the measurement properties (e.g., reliability and validity, sensitivity and specificity) of standardized tests or assessment tools you are considering using in your practice? | ○ **0%** | ○ **10** | ○ **20** | ○ **30** | ○ **40** | ○ **50** | ○ **60** | ○ **70** | ○ **80** | ○ **90** | ○ **100%** |
|  | … interpret study results obtained using statistical tests such as t-tests or chi-square tests? | ○ **0%** | ○ **10** | ○ **20** | ○ **30** | ○ **40** | ○ **50** | ○ **60** | ○ **70** | ○ **80** | ○ **90** | ○ **100%** |
|  | … interpret study results obtained using statistical procedures such as linear or logistic regression? | ○ **0%** | ○ **10** | ○ **20** | ○ **30** | ○ **40** | ○ **50** | ○ **60** | ○ **70** | ○ **80** | ○ **90** | ○ **100%** |
|  | … determine if evidence from the research literature applies to your patient’s or client’s situation? | ○ **0%** | ○ **10** | ○ **20** | ○ **30** | ○ **40** | ○ **50** | ○ **60** | ○ **70** | ○ **80** | ○ **90** | ○ **100%** |
|  | … ask your patient or client about his/her needs, values and treatment preferences? | ○ **0%** | ○ **10** | ○ **20** | ○ **30** | ○ **40** | ○ **50** | ○ **60** | ○ **70** | ○ **80** | ○ **90** | ○ **100%** |
|  | … decide on an appropriate course of action based on integrating the research evidence, clinical judgment and patient or client preferences? | ○ **0%** | ○ **10** | ○ **20** | ○ **30** | ○ **40** | ○ **50** | ○ **60** | ○ **70** | ○ **80** | ○ **90** | ○ **100%** |
|  | … continually evaluate the effect of your course of action on your patient’s or client’s outcomes? | ○ **0%** | ○ **10** | ○ **20** | ○ **30** | ○ **40** | ○ **50** | ○ **60** | ○ **70** | ○ **80** | ○ **90** | ○ **100%** |

| ○ **0%** | ○ **10** | ○ **20** | ○ **30** | ○ **40** | ○ **50** | ○ **60** | ○ **70** | ○ **80** | ○ **90** | ○ **100%** | |
| --- | --- | --- | --- | --- | --- | --- | --- | --- | --- | --- | --- |
| **No Confidence** | |  |  |  |  |  | **Completely Confident** | | | |  |

**Section 5 includes 5 questions regarding your demographic information**

**The following statements relate to your current academic status and previous academic experiences. Please circle your response.**

1. **What academic year are you currently in?**

- U1
- U2
- U3
- QY
- M1
- M2

1. **Do you hold a previous degree?**

- No
- Yes (please specify field)
  - Bachelor’s Degree in _______________
  - Master’s Degree in _________________
  - PhD in __________________________
  - Other 🡪 Please specify: _______________

1. **Do you have any previous research experience (eg. Lab technician, research assistance)**

- No
- Yes (please specify) ________________

1. **What is your current approximate cGPA (or cGPA upon graduation if M2)?**

- 2.3-3.0
- 3.0-3.3
- 3.4-3.7
- 3.8-4.0
- I prefer not to say

1. **In what age group do you belong?**

- 17-19
- 20-24
- 25-30
- 31 and over
