# Supplementary material for: Students’ attitudes and perceptions of teaching and assessment of evidence-based practice in an occupational therapy professional Master’s curriculum: a mixed methods study
Source: BMC Med Educ. 2017 Mar 27;17:64. doi: 10.1186/s12909-017-0895-2 (PMC5368912; doi:10.1186/s12909-017-0895-2)
Supplement: Supplementary file 2 — This file contains the focus group interview protocol. (DOCX 15 kb) [file 12909_2017_895_MOESM2_ESM.docx]

**Focus Group Questions**

**Focus Group Questions:** Thank you for coming today to join our discussion of EBP. We’ve invited you here today because we would like to know more about your thoughts regarding EBP and its teaching in the OT curriculum at McGill. We believe that as M2 students, your input will be valuable in helping us better understand the role of the OT program in helping you become evidence based practitioners.

We will have a discussion based on a series of questions that were informed by the answers you provided on the survey we administered last month. The session will last approximately 90 minutes. Before we begin, I would like to share a couple of ground rules. Firstly, we want you to do the talking. I will be asking questions and listening to what you have to say, but we want to hear your opinions and thoughts. Along the same lines, there are no right or wrong answers, we simply want to hear everyone’s opinion. We will also be recording today’s session so it can be reviewed at a later time by the student researchers, but wish to assure everyone that you will remain anonymous. We also ask that you respect the confidentiality of everyone else – what is said in this room stays here. We want to ensure that everyone feels comfortable sharing what they think and feel.

We will begin by asking all participants to please introduce yourselves to the facilitators. Are there any questions before we begin? *After participants introduce themselves ask:* “If it is ok with everyone, we will turn on the recorder and start now”.

Icebreaker Question: How do you define EBP?

1. In your opinion what role does EBP have in OT practice?

- - *What do you feel constitutes good application of EBP?*
  - *What does it mean to use EBP or to be an evidence based practitioner?*

2. Can you tell me a little about how has your thinking towards EBP changed since the beginning the program?

- - *What has influenced this change?*
  - *How have your attitudes towards EBP changed?*

3. In what ways has the program helped you become an evidence-based practitioner?

- - *Ideally, what should the program have done? (Examples: feedback?)*
    - *What is missing?*
  - *Which evaluation methods or teaching strategies reflect the importance of EBP?*
  - *Can you tell us about the impact or the role of your fieldwork placements on your development as an evidence based practitioner?*
  - *Do you have an example of a situation that helped you learn how to apply EBP? For example, a guest speaker, assignment or lecture?*
  - *How has your training shaped or affected your confidence in the various aspects of EBP?*
    - *What aspects are you most confident in?*

*What else could the program have done to increase your confidence?*

4. There seem to be differences in how students feel about approaching professors with questions regarding EBP. Do you have any thoughts on why some people may feel uncomfortable approaching faculty members?

5. How do you think you learn best about EBP?

- *For example, from articles, case studies, guest lectures, etc*

6. How prepared do you feel about being an evidence-based practitioner?

- *What would you need in order to feel more prepared to apply EBP as a clinician?*

Closing: Anything else in these last few minutes that you would like to share?
